# A System-Level Mechanism of Anmyungambi Decoction for Obesity: A Network Pharmacological Approach

**DOI:** 10.3390/biom11121881

**Published:** 2021-12-15

**Authors:** Dongyeop Jang, Hayeong Jeong, Chang-Eop Kim, Jungtae Leem

**Affiliations:** 1 Department of Physiology, College of Korean Medicine, Gachon University, Seongnam-si 13121, Korea; ggg5438@gachon.ac.kr (D.J.); jhyg1003@gmail.com (H.J.); 2 Research Center of Traditional Korean Medicine, College of Korean Medicine, Wonkwang University, 460, Iksan-daero, Sin-dong, Iksan 54538, Korea

**Keywords:** obesity, anmyungambi decoction, traditional Asian medicine, adipocytokine, lipolysis, insulin signaling pathway, type II diabetes mellitus, non-alcoholic fatty liver disease

## Abstract

Obesity is a low-grade systemic inflammatory disease involving adipocytokines. As though Anmyungambi decoction (AMGB) showed significant improvement on obesity in a clinical trial, the molecular mechanism of AMGB in obesity remains unknown. Therefore, we explored the potential mechanisms of action of AMGB on obesity through network pharmacological approaches. We revealed that targets of AMGB are significantly associated with obesity-related and adipocyte-elevated genes. Evodiamine, berberine, genipin, palmitic acid, genistein, and quercetin were shown to regulate adipocytokine signaling pathway proteins which mainly involved tumor necrosis factor receptor 1, leptin receptor. In terms of the regulatory pathway of lipolysis in adipocytes, norephedrine, pseudoephedrine, quercetin, and limonin were shown to affect adrenergic receptor-beta, protein kinase A, etc. We also found that AMGB has the potentials to enhance the insulin signaling pathway thereby preventing type II diabetes mellitus. Additionally, AMGB was discovered to be able to control not only insulin-related proteins but also inflammatory mediators and apoptotic regulators and caspases, hence reducing hepatocyte injury in nonalcoholic fatty liver disease. Our findings help develop a better understanding of how AMGB controls obesity.

## 1. Introduction

Obesity is one of the major global healthcare problems with rapidly increasing prevalence [1]. Obesity is regarded as a low-grade systemic inflammatory disease associated with cytokines [2,3]. Adipocytes produce and secrete the adipocytokines tumor necrosis factor-alpha (TNF-a), interleukin (IL)-6, and leptin (LEP), the levels of which are directly related to the degree of obesity [3]. Obesity affects various organ systems such as kidney, endocrine, gastrointestinal and cardiovascular systems, resulting in increasing mortality [4,5]. Given the multidimensional factors contributing to excessive fat deposition such as environmental, behavioral, physiological, social, and genetic factors [1], various attempts have been made for the treatment of obesity. Among the approaches, pharmacological treatment is a major approach in western medicine and usually aims to reduce food consumption by inhibiting appetite or craving for food. However, many of those have been criticized for their poor medication adherence [6,7]. Moreover, it was reported only about 20% of overweight individuals maintain long-term weight loss [8] Therefore, developing anti-obesity drugs showing long-term efficacy and safety is attracting growing attention. 

In traditional East Asian medicine, herbal medicine is widely used for obesity treatment [9]. Anmyungambi decoction (AMGB), composed of Ephedrae Herba (EH), Gardeniae Fructus (GF), Glycine Semen Preparata (GSP), and Phellodendri Cortex (PC), was developed and has been used for the treatment of obesity [10]. The preparation was designed to utilize the synergistic anti-obesity effect of multiple herbs while preventing the potential adverse effects of EH by combining multiple herbs. In a previous clinical study, more than half of patients treated by AMGB lost more than 5% of their body weights without severe adverse effects [10]. Despite the clinical evidence, however, the molecular mechanism of weight loss and side effect reduction in AMGB remains unknown.

Herbal medicine contains multiple compounds and is known to act on multiple targets simultaneously [11]. Therefore, it is difficult to grasp the overall mechanisms and efficacy of herbal medicine by employing conventional pharmacological analysis. Network pharmacology, an effective method to explore efficacy and mechanisms of multiple compounds at the systems level, has been widely used in exploring candidate combinations of medicinal herbs drug development and predicting possible mechanisms and adverse effects of herbal prescription [11,12,13,14,15]. Since AMGB consists of multiple herbs which are expected to synergistically affect obesity, applying network pharmacological analysis will be an effective strategy to understand the potential mechanisms of AMGB.

In this study, we tried to decipher the potential effects and mechanisms of AMGB for regulating obesity and its complications via the network pharmacological approach. First, to investigate whether AMGB can affect obesity, we analyzed the statistical significance of the overlap between potential targets of AMGB and genes related to obesity. Next, to explore which type of tissue is related to targets of AMGB, we analyzed the statistical significance of the overlap between targets of AMGB and elevated genes in adipocytes. Finally, to understand the mechanisms of AMGB for obesity, we investigated potential pathways of AMGB related to obesity.

## 2. Materials and Methods

### 2.1. Collection of Compound Information

The TM-MC (https://informatics.kiom.re.kr/compound/index.jsp (accessed on 17 November 2021)) [16] were used to obtain compounds information of herbs in AMGB. The compounds that may not affect oral administration were filtered out based on the quantitative estimate of drug-likeness (QED), drug-likeness scores based on molecular descriptors ranging from 0 to 1 [17]. The cut-off value of QED for ruling out the compounds was 0.35.

### 2.2. Construction of an Herb–Compound–Target Network

The STITCH database (http://stitch.embl.de/ (accessed on 17 November 2021)) [18] was used to obtain the potential targets of the drug-like compounds. Interactions for which the predictions are highly confident (combined score >0.7) were chosen from the predicted interactions between compounds and targets. The herb–compound–target network was built by connecting herb nodes, compound nodes, and target nodes using the drug-like compounds of herbs collected in AMGB and their potential targets.

### 2.3. Targets-Related Disease and Pathway Analysis

Gene set enrichment analysis (GSEA) based on DisGeNET (The Human Protein Atlas) [19,20] and Kyoto Encyclopedia of Genes and Genomes (KEGG) databases [21] was performed to infer the relations between potential targets of AMGB and obesity-associated (tissue-specific) genes and pathways, respectively. In GSEA, adjusted p-values and combined scores, the logarithm of the multiplication of the *p*-value and z-score of overlap between targets and gene sets, were calculated. All the enrichment analyses were conducted using Enrichr, an open-source and freely available enrichment analysis web-based tool [22]. To analyze the effects of AMGB on KEGG pathways at the herb level, we defined a combined score of an herb as a sum of combined scores of their compounds with adjusted *p*-values lower than 0.05. Moreover, to predict comprehensive effects of herbs on multiple KEGG pathways in the category level, we defined a combined score for a category of KEGG pathways as the averaged combined scores for pathways included in the category.

## 3. Results

We constructed an herb–compound–target network which consisted of 969 nodes corresponding to the herb (4), potential bioactive compounds (156), or their targets (809), and 1457 edges indicating that the compounds are contained in the herbs (between herb nodes and compound nodes) or the compounds interact with the targets (between compound nodes and target nodes).

To investigate whether AMGB and its herbs can affect obesity, we performed GSEA to estimate how much the targets of AMGB overlap with obesity-associated genes retrieved from DisGeNET. We found that the targets of AMGB and all the individual herbs were significantly overlapped with the obesity-associated genes (Table 1). Notably, obesity is the most highly ranked disease among 8066 diseases in GSEA for AMGB, GF, and EH. This result implies that AMGB can affect the progression of obesity through regulating genes related to obesity. To explore which types of tissue AMGB and its herbs target, we performed GSEA based on The Human Protein Atlas. We found that targets of AMGB were outstandingly overlapped with elevated genes in adipocytes (Table 2). Among the herbs in AMGB, EH and GF were strongly related to adipocytes (Supplementary Table S1).

**Figure 1:**
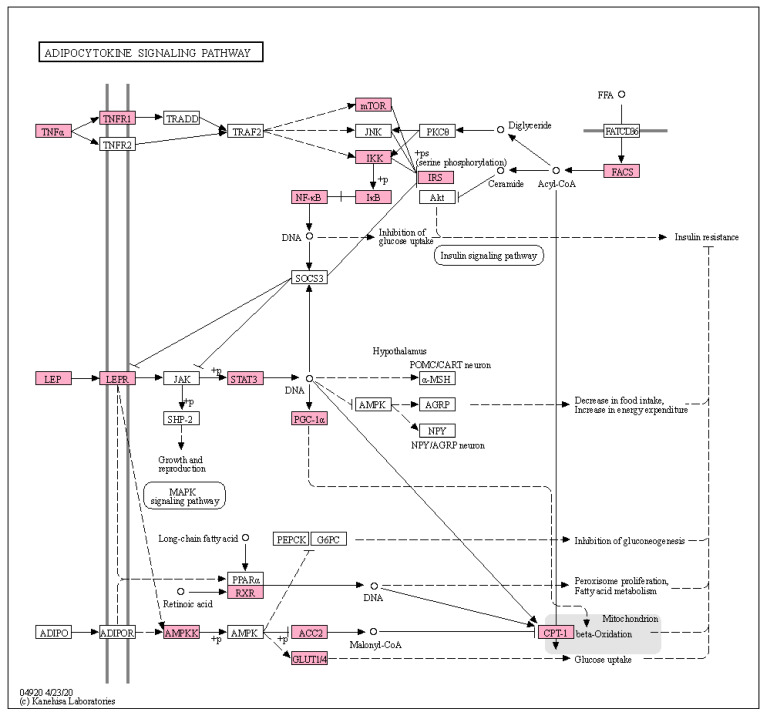
Adipocytokine signaling pathway and targets of AMGB, visualized by KEGG mapper. Boxes represent proteins that compose the adipocytokine signaling pathway. The pink-colored boxes represent targets of compounds of AMGB which are significantly associated with the adipocytokine signaling pathway.

**Figure 2:**
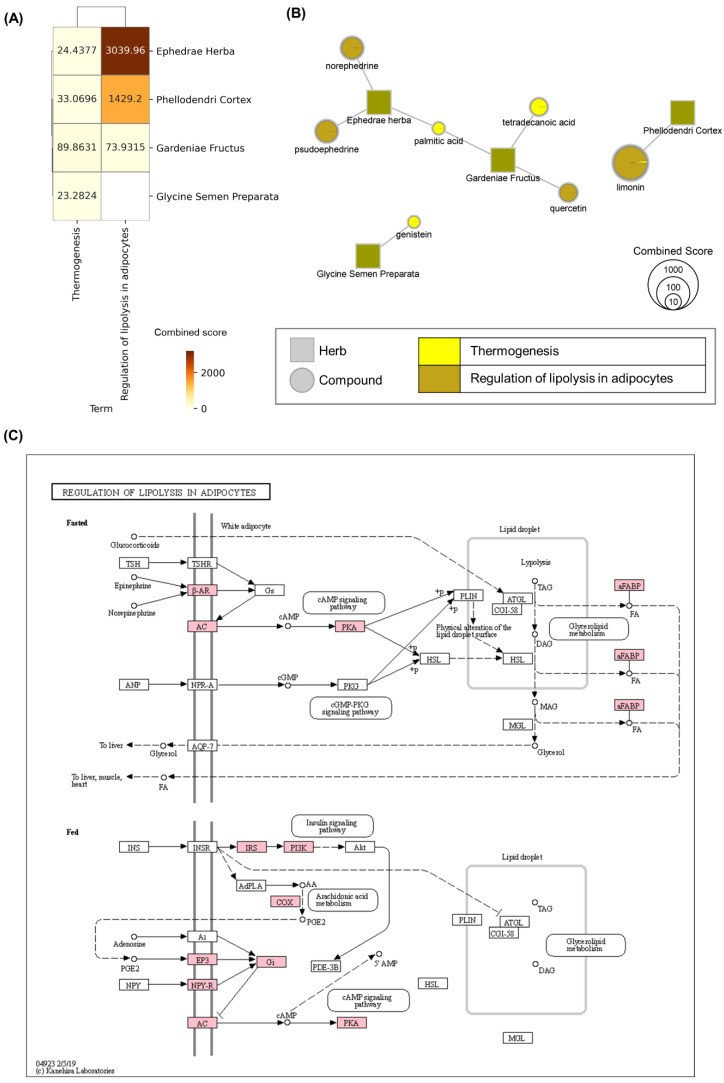
Association between herbs in AMGB and pathways related to lipolysis. (**A**) Cluster heatmap of combined scores of herbs. (**B**) Herb–compound network of AMGB, focusing on the association between compounds and pathways. (**C**) Pathway for regulation of lipolysis in adipocytes and predicted targets of AMGB, visualized by KEGG mapper. Boxes represent proteins that compose the pathway. The pink-colored boxes represent predicted targets of AMGB. All the details are the same as in Figure 1.

**Figure 3:**
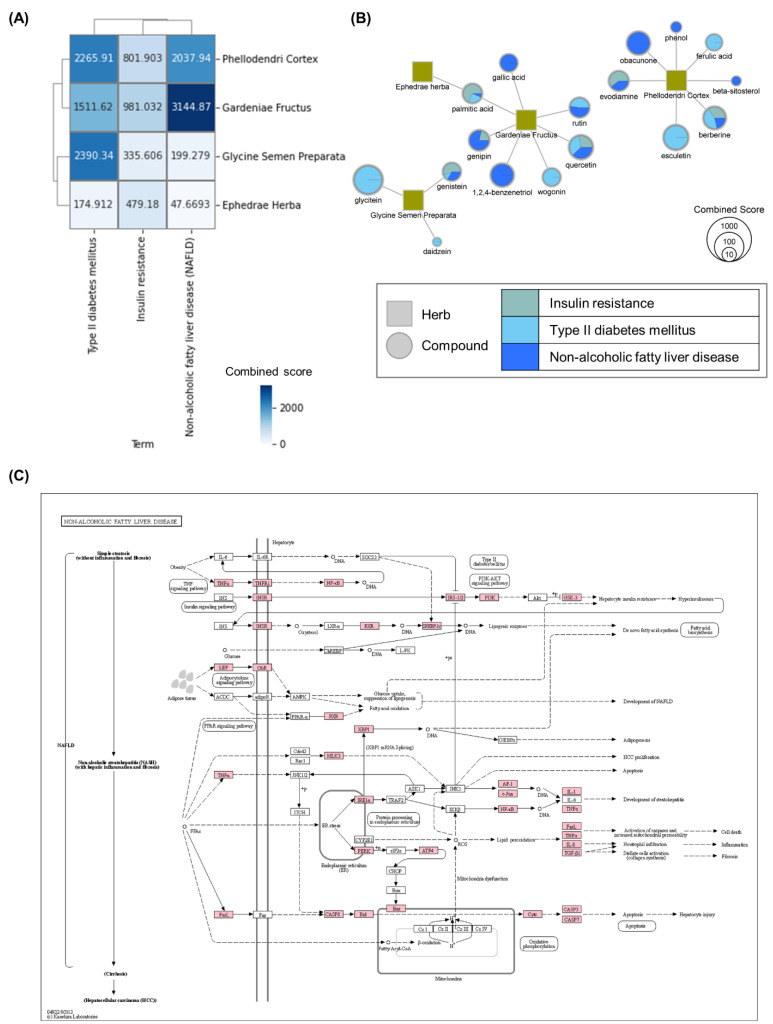
Association between herbs in AMGB and pathways related with complications resulting from obesity. (**A**) Cluster heatmap of combined scores of herbs. (**B**) Herb–compound network of AMGB, focusing on the association between compounds and pathways. (**C**) Pathway of non-alcoholic fatty liver disease and predicted targets of AMGB, visualized by KEGG mapper. Boxes represent proteins that compose the pathway. The pink-colored boxes represent predicted targets of AMGB. All the details are the same as in Figure 2.

To decipher the mechanisms of AMGB for obesity, we performed GSEA for obesity-related KEGG pathways. First, we focused on the association between targets of compounds in AMGB and adipocytokine signaling pathway since the adipocytokine signaling pathway including leptin is considered the main pathophysiology of obesity at the molecular level [23]. We found that targets of evodiamine, berberine, genipin, palmitic acid, genistein, and quercetin were significantly overlapped with genes in the adipocytokine signaling pathway (Table 3). AMGB was found to target multiple proteins in the adipocytokine signaling pathway including TNF, LEP, and adiponectin (Figure 1). Especially, genistein and quercetin target the TNF receptor and leptin receptor, respectively. These results suggest that AMGB can affect the pathophysiology of obesity via regulating the adipocytokine signaling pathway. We next investigated the association between AMGB and pathways including regulation of lipolysis in adipocyte and thermogenesis. EH, PC, and GF in AMGB showed significant relation to both the regulation of lipolysis in adipocyte and thermogenesis, and GSP was significantly associated with thermogenesis. In the case of regulation of lipolysis in adipocytes, EH showed the highest combined score, suggesting that EH plays a key role in regulating lipolysis in adipocytes (Figure 2A). Norephedrine and pseudoephedrine (in EH), limonin (in PC), and quercetin (in GF) were found to be significantly associated with the regulation of lipolysis in adipocytes, indicating that these compounds contribute to lipolysis in the adipocyte. Meanwhile, palmitic acid, tetradecanoic acid (in GF), limonin (in PC), and genistein (in GSP) were significantly associated with thermogenesis, suggesting the possibility that these compounds facilitate thermogenesis in brown adipocytes consuming fatty acids (Figure 2B). In the regulation of lipolysis in adipocytes, receptors including beta-adrenergic receptor and their downstream signaling pathways were shown to be targeted by AMGB (Figure 2C). Finally, we investigated whether and how AMGB can regulate complications of obesity including insulin resistance, type II diabetes mellites, and non-alcoholic fatty liver disease (NAFLD). Targets of every herb in AMGB were found to be significantly overlapped with proteins that compose pathways about complications of obesity, suggesting that every herb in AMGB could regulate complications of obesity. Especially, PC and GF showed strong relations to NAFLD, while PC and GSP were found to be intensively related to type 2 diabetes mellitus (Figure 3A). Among compounds in GF, 1,2,4-Benzenetriol showed the highest combined score on NAFLD. Among compounds in GSP and PC, glycitein and esculetin showed the highest combined scores on type II diabetes mellitus. In order to identify how AMGB can control the development of NAFLD, we investigated targets of AMGB which were overlapped with proteins that compose the pathway of NAFLD. As a result, targets of AMGB were found to be associated with the TNF signaling pathway, insulin signaling pathway, and peroxisome proliferator-activated receptors (PPAR) signaling pathway which contributes to the development of NAFLD [24] (Figure 3C).

In summary, our results imply that EH, PC, GF, and GSP in AMGB can regulate the adipocytokine signaling pathway, promote lipolysis, and inhibit the progress of complications of obesity. We also suggest that these regulations are based on synergistic effects of various bioactive compounds of herbs in AMGB.

## 4. Discussion

We investigated the potential efficacy and mechanism of action of AMGB in the treatment of obesity using network pharmacological techniques. Our findings imply that AMGB and its compounds have the potential to not only promote weight loss by modifying lipolysis in adipocytes and adipocytokine signaling pathways but also to avoid potential obesity-related comorbidities.

AMGB is composed of EH and PC, which has an anti-obesity effect, as well as GH and GSP, which mitigate EH’s adverse effects and enhance its effect. EH is one of the most important medicinal herbs for the treatment of obesity [25,26]. It is well established that ephedrine accounts for approximately 30–90% of EH’s total alkaloids [27]. Ephedrine induces lipolysis, suppresses appetite, increases heat production, promotes energy consumption, and inhibits cholesterol absorption [27]. However, it causes adverse effects on the cardiovascular system and nervous systems such as palpitation, hypertension, and tachycardia [28]. Meanwhile, GF and GSP that are collectively referred to as the Zhizichi decoction are widely used to treat psychological symptoms such as anxiety and sleeplessness [29]. GF and GSP are added as component herbs of AMGB to alleviate the psychological symptoms associated with sympathetic nerve hyperstimulation produced by EH [10]. PC is another medicinal herb for weight loss. It involves inhibition of adipocyte development and the mRNA and protein expression of PPARγ [30]. Indeed, AMGB significantly alleviated obesity in a previous clinical study with no severe adverse effects reported. [10]. However, the mechanisms of action of AMGB in the treatment of obesity are uncertain.

Abnormal signaling of adipocytes aggravates obesity and induces impaired organ communications and metabolic abnormalities in multiple tissues thereby constituting a critical pathological component in the development of metabolic disease [22,30,31]. We discovered that evodiamine, berberine, genipin, palmitic acid, genistein, and quercetin can modulate proteins in the adipocytokine signaling pathway including tumor necrosis factor receptor 1 (TNFR1), signal transducer, and activator of transcription 3 (STAT3), leptin receptor (LEPR), glucose transporter type 1/4 (GLUT1/4), nuclear factor kappa B (NF-kB), inhibitor of NF-kB kinase (IKK), and mammalian target of rapamycin (mTOR), implying that these compounds regulate the dysfunctional signaling of adipocytes. TNF-α is an inflammatory cytokine that has been linked to the development of insulin resistance. Interaction between TNF-α and NF-kB signaling activates IKK-β and increases oxidative stress, resulting in endothelial dysfunction in type 2 diabetes [31]. Meanwhile, LEPR is found on brain neurons involved in energy intake, and delivery of leptin directly into the brain decreases food intake [32]. Leptin resistance is characterized by decreased satiety, excessive food consumption, and an increase in total body mass [33] and a significant issue in obesity. While controversial, enhanced STAT3 activation is regarded to ameliorate leptin resistance [34]. Indeed, STAT3 activity was considerably increased in the hypothalamus of diet-induced obesity mice, accompanied by lower pro-opiomelanocortin (POMC) expression and abnormal metabolic physiological behaviors, implying that increased STAT3 activity negatively affected leptin-mediated POMC expression in diet-induced obesity mice [34]. Moreover, excess STAT3 activity inhibited POMC expression in the hypothalamus of diet-induced obesity mice, implying that STAT3 inhibition may promote leptin signaling [35]. GLUT1/4 promotes glucose transport across plasma membranes [36], and PPAR-γ inhibitors increase expression levels of GLUT1/4 thereby promoting glucose transports in muscle and liver [37]. mTOR signaling is the most essential intracellular mechanism that coordinates local nutrition availability and systemic energy status, and its dysregulation is related to obesity and type 2 diabetes [38]. By serine phosphorylation and insulin receptor substrate 1 (IRS1) inhibition by mTOR, activation of the mTOR signaling pathway has been shown to reduce insulin sensitivity [39]. Additionally, it has been reported that berberine, evodiamine, genistein, and quercetin modulate the insulin signaling pathway by inhibiting mTOR, IRS1 serine phosphorylation, and up-regulating the phosphorylation of insulin receptor and protein kinase B [40,41,42,43]. Moreover, berberine, evodiamine, and quercetin inhibit STAT3 signaling, suggesting that these AMGB improve leptin resistance by regulating STAT3 signaling [44,45,46]. Taken together, regulating abnormal signaling of adipocytokines may be one of the main mechanisms for AMGB to play a role in the treatment of obesity.

Additionally, we found that norephedrine, pseudoephedrine (in EH), quercetin (in GF), and limonin (in PC) in AMGB affect multiple proteins involved in the regulation of lipolysis in adipocytes, including adrenergic receptor-beta (beta-AR), adenylate cyclase (AC), protein kinase A (PKA), prostaglandin E receptor 3 (EP3), neuropeptide Y receptor type 1 (NPY-R), IRS, phosphoinositide 3-kinase (PI3K), cyclooxygenase (COX), and fatty acid-binding proteins (FABPs). We note that these targets are associated with both fasted and fed states. In the fasted state, activated beta-AR promotes the cyclic adenosine monophosphate (cAMP) signaling pathway via AC and PKA [47], and FABPs regulate lipid storage and lipid-mediated gene expression [48]. In animal models, it was found that FABP4 inhibitors ameliorate insulin resistance, diabetes mellitus, fatty liver disease, and atherosclerosis, implying that inhibition of FABP4 may be a therapeutic strategy for metabolic syndrome [49]. Meanwhile, the fed state activates the insulin signaling pathway by IRS and PI3K, stimulating EP3 and NPY-R via downregulated cAMP signaling pathway by inhibition of AC. The PI3K/AKT signaling pathway is essential for optimal metabolism and its dysregulation results in the development of obesity [50]. Ephedrine in EH, which is structurally similar to ephedrine and pseudoephedrine, stimulates thermogenesis in brown adipocytes via beta-AR [45] resulting in lipolysis in brown adipocytes. Also, limonin in PC inhibits upregulated phosphorylation of PKA, resulting in modulating the cAMP signaling pathway [51]. Moreover, quercetin suppressed the protein expression levels of PI3K [52,53,54] Our findings suggest that AMGB stimulates lipolysis in adipocytes mediating the cAMP signaling pathway and related proteins.

Insulin resistance, type 2 diabetes mellitus, and NAFLD are major complications induced by obesity [55]. Obesity induced by fat dietary results in beta cell hyperplasia but not in enhanced insulin release in mice [56]. Lipid accumulation aggravates excess of circulating free fatty acids leading to destroy functions of β-cell [57]. β-cell dysfunction linked with decreases in insulin releases is likely to result in decreased insulin action in the brain, as well as weight gain and exacerbation of insulin resistance [58]. Impairment of insulin action is associated with hepatic steatosis, which results in increased free fatty acid transport to the liver [59,60]. The dysregulation of the PI3K/AKT signaling pathway also can lead to insulin resistance, type 2 diabetes, and NAFLD [50]. Treatments targeting PI3K/AKT pathways improve β-cell functionality [61], glucose uptake [62], insulin secretion [57], insulin sensitivity [63], and hepatic gluconeogenesis [64]. Our findings indicate that AMGB has the potential to control TNF-alpha, mTOR, extracellular signal-regulated kinases (ERK), IRS, and PI3K, consequently improving the insulin signaling pathway and preventing type II diabetes mellitus ( Supplementary Figure S1). Additionally, we emphasize that AMGB does not modulate a few targets related to the NAFLD pathway but can exert therapeutic effects by comprehensively regulating the components of several pathways related to the disease. Specifically, AMGB can regulate not only insulin-related proteins, but also inflammatory mediators (including TNF-alpha, IL-1, and IL-8) and apoptotic regulators and caspases (including Bcl-2-associated X protein (BAX), CASP3 (caspase 3), CASP8, and CASP7), hence preventing hepatocyte injuries in NAFLD.

These findings demonstrate how our study strategy enables us to understand the effects of drugs in a holistic manner, rather than focusing just on a specific pathway. More experiments are needed to verify the mechanism of action of AMGB for obesity.

## 5. Conclusions

We explored the potential mechanisms of action of AMGB on obesity through network pharmacological approaches. We discovered that predicted targets of AMGB were significantly associated with obesity-related and adipocyte-elevated genes. AMGB was found to be related to the adipocytokine signaling pathway, implying that AMGB may be beneficial in preventing obesity via regulating insulin and leptin signaling pathways. It was also found that multiple bioactive compounds are significantly related to the regulation of lipolysis in adipocytes and thermogenesis, implying that AMGB can stimulate lipolysis via the compounds. Moreover, AMGB was found to be significantly related to insulin resistance, type II diabetes mellitus, and NAFLD, suggesting that AMGB may be able to regulate diabetes and NAFLD resulting from obesity. Our findings contribute to the understanding of the mechanism by which AMGB regulates obesity.

## Figures and Tables

**Table 1:** GSEA of AMGB and individual herbs for obesity.

Drug	Rank	Overlap	*p*-Value (Adjusted)	Odds Ratio	Combined Score
Anmyungambi decoction (AMGB)	1	343/1961	6.00 × 10^−135^	7.99	2542.58
Phellodendri Cortex (PC)	4	125/1961	3.08 × 10^−49^	7.80	928.50
Gardeniae Fructus (GF)	1	247/1961	6.43 × 10^−104^	8.76	2159.18
Glycine Semen Preparata (GSP)	42	84/1961	1.45 × 10^−44^	12.98	1374.75
Ephedrae Herba (EH)	1	168/1961	2.35 × 10^−69^	8.49	1415.37

**Table 2:** Top 10 tissues targeted by compounds of AMGB.

Term	Overlap	*p*-Value (Adjusted)	Odds Ratio	Combined Score
Adipocyte	26/181	1.59 × 10^−6^	4.08	72.32
Smooth muscle	38/363	3.59 × 10^−6^	2.86	46.43
Adrenal cortex	14/106	1.88 × 10^−3^	3.66	33.90
Liver	52/618	1.40 × 10^−5^	2.26	32.69
Hypothalamus	12/97	6.14 × 10^−3^	3.38	25.48
Bronchial epithelial cells	25/280	3.06 × 10^−3^	2.37	20.28
Fetal liver	13/126	1.56 × 10^−2^	2.76	17.44
Placenta	32/405	3.40 × 10^−3^	2.08	17.19
Lymphnode	5/37	9.82 × 10^-2^	3.72	15.41
Cardiac myocytes	22/273	1.56 × 10^-2^	2.11	13.40

**Table 3:** GSEA of compounds in AMGB for adipocytokine signaling pathway. TNF: tumor necrosis factor, IRS1: insulin receptor substrate 1, STAT3: signal transducer and activator of transcription 3, STK11: serine/threonine kinase 11, SLC2A4 solute carrier family 2 member 4, NFKBIB: NF-kappa-B inhibitor beta, CPT1A: carnitine palmitoyltransferase 1A, RXRA: retinoid X receptor-alpha, ACSL1: acyl-CoA synthetase long-chain family member 1, ACSBG1: acyl-CoA synthetase bubblegum family member 1, ACACB: acetyl-CoA carboxylase beta, PPARGC1A: peroxisome proliferator-activated receptor gamma coactivator 1-alpha, RELA: transcription factor p65, MTOR: mammalian target of rapamycin, LEP: leptin, SLC2A1: solute carrier family 2 member 1, TNFRSF1A: TNF receptor superfamily member 1A, NFKBIA: NF-kappa-B inhibitor alpha, CHUK: conserved helix-loop-helix ubiquitous kinase, LEPR: leptin receptor.

Drug	Herb Including	Overlap	*p*-Value (Adjusted)	Odds Ratio	Combined Score	Genes
Evodiamine	PC	2/69	1.20 × 10^−3^	99.13	796.46	IRS1, STAT3
Berberine	PC	5/69	1.01 × 10^−5^	31.70	432.97	STK11, STAT3, SLC2A4, TNF, NFKBIB
Genipin	GF	2/69	3.04 × 10^−3^	45.74	307.73	IRS1, STAT3
Palmitic acid	EH, GF	12/69	2.49 × 10^−8^	12.70	257.17	CPT1A, RXRA, IRS1, ACSL5, ACSL4, ACSBG1, ACSL3, TNF, ACACB, PPARGC1A, RELA, MTOR
Genistein	GSP	6/69	3.43 × 10^−5^	15.21	183.54	CPT1A, LEP, STAT3, SLC2A1, TNF, TNFRSF1A
Quercetin	GF	6/69	7.56 × 10^−5^	12.82	142.69	NFKBIA, CHUK, SLC2A1, LEPR, SLC2A4, TNF

## Data Availability

The datasets used and/or analyzed during the current study are available from the corresponding author on reasonable request.

## Supplementary Materials

The following are available online at https://www.mdpi.com/article/10.3390/biom11121881/s1, Figure S1: Pathway of type II diabetes mellitus and predicted targets of AMGB, visualized by KEGG mapper, Table S1: Enrichment analysis of tissues by the targets of compounds of Phellodendri Cortex, Gardeniae Fructus, Glycine Semen Preparata, and Ephedrae Herba.
