# Supplementary material for: A System-Level Mechanism of Anmyungambi Decoction for Obesity: A Network Pharmacological Approach
Source: Biomolecules. 2021 Dec 15;11(12):1881. doi: 10.3390/biom11121881 (PMC8699029; doi:10.3390/biom11121881)
Supplement: Supplementary file 1 [file biomolecules-11-01881-s001.zip › biomolecules-1490612-supplementary.pdf]

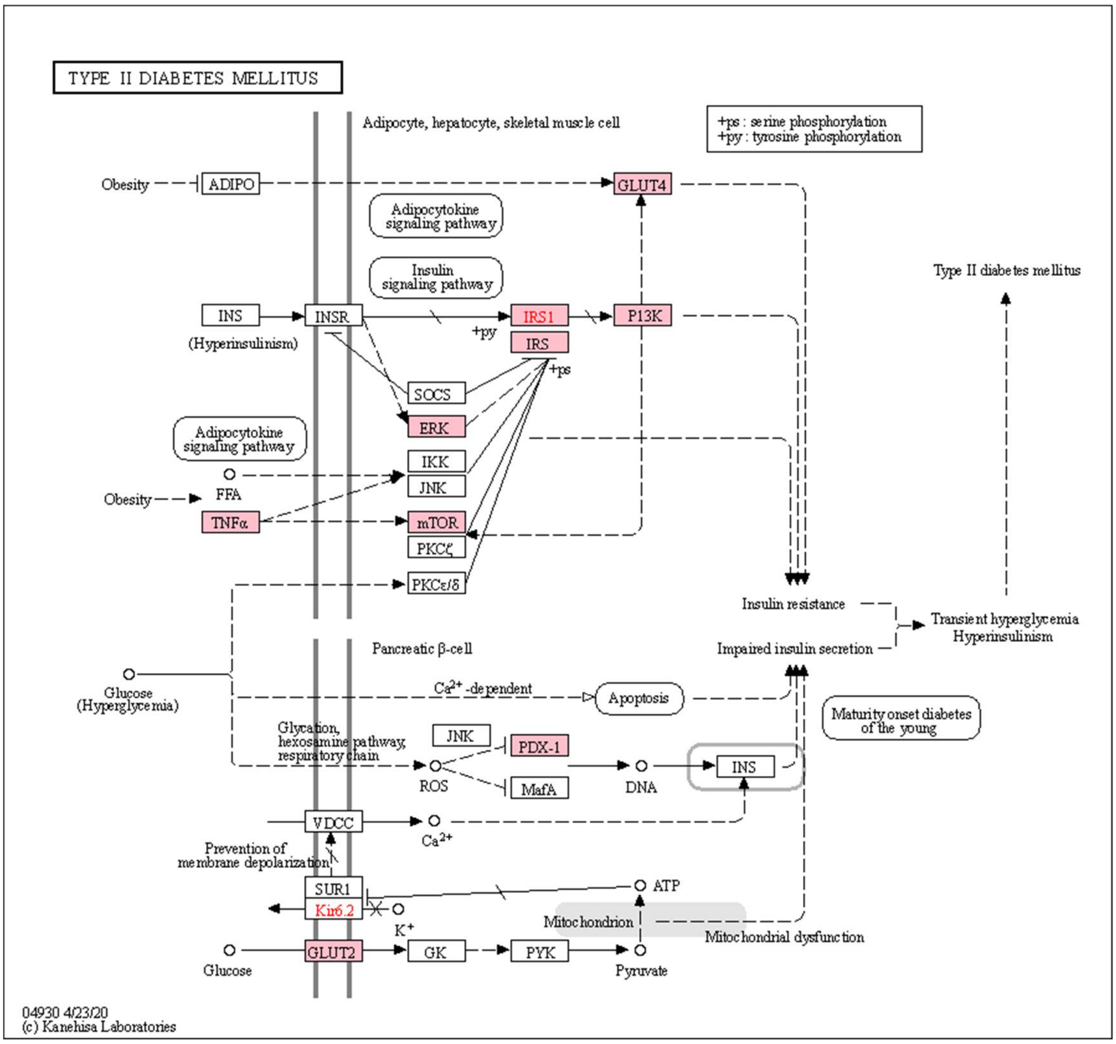


**Supplementary figure S1.** Pathway of type II diabetes mellitus and predicted targets of AMGB, visualized by KEGG mapper. Boxes represent proteins that compose the pathway. The pink-colored boxes represent predicted targets of AMGB. All the details are the same as in Figure 2.

**Supplementary Table S1.** Enrichment analysis of tissues by the targets of compounds of Phellodendri Cortex, Gardeniae Fructus, Glycine Semen Preparata, and Ephedrae Herba. Only the results with P-values < 0.05 are shown.

| Herb | Term | Overlap | P-value (adjusted) | Odd ratio | Combined Score |
|---|---|---|---|---|---|
| Phellodendri Cortex | Lymph node | 5/37 | 0.0056 | 11.15 | 97.46 |
| Phellodendri Cortex | Adrenal cortex | 8/106 | 0.0056 | 5.87 | 52.67 |
| Gardeniae Fructus | Adipocyte | 21/181 | 1.61E-06 | 4.89 | 86.55 |
| Gardeniae Fructus | Smooth muscle | 29/363 | 8.14E-06 | 3.26 | 50.09 |
| Gardeniae Fructus | Liver | 40/618 | 8.43E-06 | 2.62 | 39.13 |
| Gardeniae Fructus | Adrenal cortex | 11/106 | 2.43E-03 | 4.25 | 37.82 |
| Gardeniae Fructus | Fetal liver | 12/126 | 2.43E-03 | 3.86 | 33.88 |
| Gardeniae Fructus | Hypothalamus | 9/97 | 1.08E-02 | 3.74 | 25.06 |
| Gardeniae Fructus | Small intestine | 16/220 | 4.24E-03 | 2.89 | 23.18 |
| Gardeniae Fructus | Bronchial epithelial cells | 18/280 | 7.01E-03 | 2.53 | 18.66 |
| Gardeniae Fructus | Cardiac myocytes | 17/273 | 1.08E-02 | 2.44 | 16.34 |
| Glycine Semen Preparata | Placenta | 11/405 | 0.0107 | 4.02 | 34.38 |
| Glycine Semen Preparata | Liver | 13/618 | 0.0168 | 3.11 | 23.01 |
| Glycine Semen Preparata | CD105+ Endothelial | 9/370 | 0.0296 | 3.55 | 22.81 |
| Ephedrae Herba | Hypothalamus | 10/97 | 0.000412 | 6.33 | 72.19 |
| Ephedrae Herba | Adipocyte | 15/181 | 0.0000918 | 5.03 | 68.36 |
| Ephedrae Herba | Fetal liver | 9/126 | 0.00761 | 4.22 | 31.94 |
| Ephedrae Herba | Smooth muscle | 17/363 | 0.00761 | 2.72 | 21.33 |
| Ephedrae Herba | Liver | 24/618 | 0.00761 | 2.26 | 17.36 |
